# 

**Published:** 1939

**Authors:** 


### CONTENTS.

| | Page |
|---|---|
| **Diet in Pregnancy.** By J. A. Nixon, C.M.G., M.D., F.R.C.P. .. | 165 |
| **The Prevention and Treatment of Prolapse.** By H. J. Drew Smythe, M.D., M.S., F.R.C.S. F.R.C.O.G. .. .. .. .. | 177 |
| **Basal Narcosis.** By E. G. Bradbeer, M.R.C.S., L.R.C.P., D.A. .. | 181 |
| **An Investigation into the Mental State of the Parents and Sibs of 1,050 Mentally Defective Persons.** By Richard J. A. Berry, M.D., F.R.C.S., F.R.S.E. .. .. .. .. .. .. .. | 189 |
| **Reviews of Books** .. .. .. .. | 201 |
| **Editorial Notes** .. .. .. .. .. .. .. .. | 208 |
| **Obituaries:** A. L. Flemming, M.B., Ch.B. .. .. .. .. | 212 |
| F. J. A. Mayes, M.R.C.S., L.R.C.P. .. | 215 |
| Mrs. R. G. Burden .. .. .. .. .. | 216 |
| **Meetings of Societies** .. .. .. .. .. .. .. | 217 |
| **The Medical Library of the University of Bristol** .. .. | 219 |
| **Publications Received** .. .. .. .. .. .. .. | 220 |
| **Local Medical Notes** .. .. .. .. .. .. .. | 222 |

